# EMBEDDING A PHYSICIAN REFERRAL PATHWAY TO LINK PEOPLE WITH DEMENTIA TO COMMUNITY SERVICE PROGRAMS

**DOI:** 10.1093/geroni/igad104.2407

**Published:** 2023-12-21

**Authors:** Michael Lepore, Stephani Shivers, Deanna Curbeam

**Affiliations:** University of Maryland School of Nursing, Baltimore, Maryland, United States; CaringKind, New York, New York, United States; University of Maryland, Baltimore School of Nursing, Baltimore, Maryland, United States

## Abstract

Dementia care quality and value can be enhanced by lateral integration between health systems (HS) and community-based organizations (CBOs), however clinical providers are often unaware of CBO programs. HS could heighten access to CBO programs among people living with dementia (PLWD) by recognizing CBOs as extended care teams, streamlining referrals, and facilitating information exchange through electronic health records (EHRs). Catalyzing CBO and HS integration, we piloted physician referral to a CBO for virtual delivery of Cognitive Stimulation Therapy (CST) in an embedded pragmatic trial. CST, a 14-session group intervention for PLWD, has been shown to benefit participant cognition and other outcomes, yet CST access is limited partially due to the risks and costs of in-person gathering. To enhance access, we trialed a version of virtual CST (vCST) and assessed patient follow-through on physician vCST referrals. Participating sites–one primary care clinic and one neurological specialty clinic—identified eligible PLWD via EHR (e.g., cognitive score indicating mild cognitive impairment to moderate dementia) and clinicians verified eligible PLWD appropriateness for vCST. Clinician-verified PLWD were randomized to intervention (referral, n=56) and control (no referral) groups, and clinicians provided vCST referrals. Results show the percentage of PLWD who followed through on (‘accepted’) the vCST referral. Similarities and differences—across demographics, cognitive scores, and referral sources—between PLWD who accepted versus declined referral are described. Future research will assess conversion rates from vCST referral to program completion, the completeness of outcome measures captured from EHRs, and the impact of vCST on cognitive scores.

